# Difference of Clinical Manifestation Between Infection and Colonization of Pseudomonas aeruginosa Among Patients With Community-Acquired Pneumonia

**DOI:** 10.7759/cureus.73298

**Published:** 2024-11-08

**Authors:** Shoma Hirota, Akihiko Goto, Hisayuki Shuto, Kosaku Komiya

**Affiliations:** 1 Respiratory Medicine and Infectious Diseases, Oita University Faculty of Medicine, Yufu, JPN; 2 Respiratory Medicine, Tenshindo Hetsugi Hospital, Oita, JPN

**Keywords:** antimicrobial management, antimicrobial resistance, broad-spectrum antibiotics, p. aeruginosa, pneumonia

## Abstract

Background: Distinguishing *Pseudomonas aeruginosa* infection and colonization from respiratory samples is challenging. We aimed to determine useful markers for differentiating *P. aeruginosa* infection from colonization in community-acquired pneumonia (CAP) patients.

Methods: We included CAP patients in whom *P. aeruginosa *was isolated from sputum but were not initially treated with *P. aeruginosa-*targeting antibiotics. Patients cured with antibiotics not targeting *P. aeruginosa* were defined as colonization cases, and those unresponsive to antibiotics not targeting *P. aeruginosa* and cured with antibiotics targeting *P. aeruginosa* were defined as infection cases.

Results: Of 299 CAP patients, 203 (68%) were treated with antibiotics not targeting *P. aeruginosa* in their initial regimen. After excluding 73 of 203 patients who were not cured with antibiotics targeting *P. aeruginosa *in subsequent regimens, 17 and 113 were classified as infection and colonization cases, respectively. Systolic blood pressure in the infection group was significantly lower than that in the colonization group (odds ratio = 0.971, 95% confidence interval: 0.946-0.996); no other significant differences were observed.

Conclusions: Low systolic blood pressure might be a useful marker for distinguishing isolated *P. aeruginosa *that need to be targeted from those that do not need to be targeted. However, interventional research is required to validate our study results.

## Introduction

Antimicrobial resistance (AMR) is a serious concern worldwide. In 2015, the World Health Organization developed a global action plan advocating for the appropriate use of antibiotics [1]. Inappropriate use of antibiotics can lead to the emergence of AMR; therefore, this action plan focused on optimizing the use of antibiotics [2]. As the extent of antibiotic use and the isolation rate of drug-resistant organisms are positively correlated [3], indications for the requirement of antibiotics should be carefully determined. Pneumonia is a major infectious disease that causes morbidity and mortality, especially among older adults. Proper use of broad-spectrum antibiotics for patients with pneumonia is a significant challenge that needs to be addressed immediately. In particular, the indications for antimicrobial treatment targeting methicillin-resistant *Staphylococcus aureus *(MRSA) or *Pseudomonas aeruginosa* are yet to be determined, although some scoring systems have been suggested to evaluate risk factors [4,5].

The 2001 American Thoracic Society (ATS) guidelines for the management of adults with community-acquired pneumonia (CAP) list advanced age, structural lung disease, immune-suppressive illness, use of broad-spectrum antibiotics for seven days in the past month, and malnutrition as risk factors for drug-resistant bacteria isolation [4]. In 2005, healthcare-associated pneumonia (HCAP), a new concept of pneumonia, was proposed by ATS and the Infectious Diseases Society of America (IDSA) [5]. Individuals are considered to have HCAP if they were hospitalized in an acute care hospital for two or more days within 90 days of infection, lived in a long-term care facility, and received intravenous antibiotic therapy within the past 30 days from the onset of the current infection. These guidelines recommended the use of broad-spectrum antibiotics for patients with HCAP due to the high frequency of drug-resistant pathogen isolation. However, the current HCAP definition does not accurately identify the risk of resistant pathogens [6]. Therefore, this concept was determined to be inapplicable to treat pneumonia in hospital-acquired pneumonia (HAP) guidelines 2016 and CAP guidelines 2019 [7,8]. Instead, the current CAP guidelines describe that the most consistently strong individual risk factors for MRSA or *P. aeruginosa* infection are prior isolation of these organisms from respiratory samples, regardless of disease severity, as well as recent hospitalization and exposure to parenteral antibiotics [8].

Nevertheless, physicians have experienced patients with CAP in whom *P. aeruginosa* was isolated from respiratory samples to be cured by treatment with antibiotics not targeting *P. aeruginosa*. In such occasions, isolated *P. aeruginosa* would have denoted colonization but not infection in the lungs. We have previously shown that treatment with antibiotics targeting *P. aeruginosa* did not significantly improve the in-hospital survival rate in older patients with CAP in whom *P. aeruginosa* was isolated from respiratory samples [9]. As *P. aeruginosa* can colonize the lower airway, especially among people with chronic respiratory diseases, it is challenging to distinguish infection from colonization of pathogens isolated from respiratory samples. Although some researchers have been focusing on the change of genetic patterns from colonization to infection [10,11], research evaluating clinical manifestation between colonization and infection is scarce. A previous study used Gram staining to identify causative pathogens in patients with pneumonia [12], but its accuracy has not been fully elucidated. Since a gold standard is lacking to differentiate between infection and colonization upon isolation of pathogens, the final diagnosis in clinical settings may be determined through the antibiotic response. For example, if *P. aeruginosa* was isolated from a patient successfully cured with antibiotics not targeting *P. aeruginosa*, the isolated *P. aeruginosa *would not need to be targeted, possibly indicating colonization, whereas if a patient was unresponsive to antibiotics not targeting *P. aeruginosa *but was subsequently cured after treatment with antibiotics targeting *P. aeruginosa*, the isolated *P. aeruginosa *would need to be targeted, possibly indicating infection [13]. Although this determination is retrospective, it seems to be more accurate in distinguishing infection from colonization. Therefore, we retrospectively classified cases in which *P. aeruginosa *was isolated from respiratory samples into colonization and infection groups based on antibiotic response. This study aimed to determine useful markers for differentiating isolated *P. aeruginosa* that needs to be targeted from *P. aeruginosa* that does not need to be targeted among patients with CAP in whom *P. aeruginosa* was isolated from respiratory samples.

This article was previously presented as an abstract at the 98th Annual Meeting of the Japanese Association for Infectious Diseases from June 27 to 29, 2024.

## Materials and methods

Patients and study design

This retrospective cohort study was conducted at two community hospitals: Bungo-Ohno City Hospital in Bungo-Ohno with 199 beds in Japan and Tenshindo Hetsugi Hospital with 188 beds in Oita, Japan. CAP was diagnosed according to the ATS/IDSA guidelines [14]. Pneumonia was diagnosed based on the clinical signs and symptoms of patients and the detection of infiltrates using chest radiography or computed tomography. We retrospectively analyzed patients aged 20 years or older who were admitted to the abovementioned two hospitals for CAP between 2009 and 2022 and in whom *P. aeruginosa* was isolated from respiratory samples at the time of admission but were not treated with antibiotics targeting *P. aeruginosa* in their first regimen. Patients in whom *P. aeruginosa* was not isolated from respiratory samples and those who were treated with antibiotics targeting *P. aeruginosa* in their first regimen even in outpatient settings were excluded from this study. Patients who were successfully cured with antibiotics not targeting *P. aeruginosa* were defined as “colonization cases.” Patients who did not respond to antibiotics not targeting* P. aeruginosa* and were cured with antibiotics targeting *P. aeruginosa* were defined as “infection cases.” Patients treated with antibiotics targeting *P. aeruginosa* in their initial regimen and those not cured with antibiotics targeting *P. aeruginosa* in the second or subsequent regimens were excluded from this study. Antibiotics available in Japan targeting *P. aeruginosa* included pazufloxacin, meropenem (MEPM), biapenem, doripenem, piperacillin (PIPC), tazobactam/piperacillin (TAZ/PIPC), sulbactam/cefoperazone, ciprofloxacin, levofloxacin (LVFX), ceftazidime, cefepime, and amikacin [15]. If the isolated *P. aeruginosa* was resistant to these antibiotics, we classified the patient into the group treated with antibiotics not targeting *P. aeruginosa*.

We obtained patient data, including sex; age; and body mass index; underlying diseases, including chronic obstructive pulmonary disease, cardiac diseases, and diabetes mellitus; laboratory data, including white blood cell (WBC) count, neutrophil count, hemoglobin level, alanine aminotransferase (ALT) level [16], C-reactive protein level (CRP), and albumin level; and the presence of respiratory failure, from available clinical records. Respiratory failure was defined as an oxygen saturation of less than 90% without supplemental oxygen. The estimated glomerular filtration rate was obtained using serum creatinine levels and age [17]. These data and the presence of respiratory failure were recorded on the day of admission. The number of *P. aeruginosa* colonies was described on a 4-point scale. Neutrophil count or phagocytic features were not routinely evaluated in these hospitals. Routine collection of patients’ data and information regarding their examination performance is recommended when patients with CAP are admitted to these hospitals. The CAP guidelines by ATS/IDSA recommend that prior isolation of *P. aeruginosa *from respiratory samples is considered a risk factor for *P. aeruginosa* infection. However, information regarding prior isolation status would be imprecise because the elderly patients had histories of admission to different clinics or hospitals. Moreover, the aim of this study was to differentiate between colonization and infection when *P. aeruginosa* was isolated from respiratory samples. The guidelines’ recommendation seems to denote the risk for *P. aeruginosa* isolation but probably not an infection. Therefore, we did not collect the information regarding the prior isolation status.

This study was in accordance with the ethical guidelines of the 1975 Declaration of Helsinki and was approved by the ethical committee of Oita University (approval number 2537; approval date June 12, 2023). Due to the retrospective nature of this study, the committee waived the need for informed consent. The corresponding author has full access to all data pertaining to this study and holds final responsibility for the decision to submit it for publication. Some patients included in this study were also enrolled in previous studies [9,18-20].

Statistical analyses

Statistical analyses were performed using the Statistical Package for the Social Sciences software, version 28 (IBM Corp., Armonk, NY). Continuous variables are expressed as median (interquartile range), whereas categorical variables are presented as numbers and percentages. The confidence interval in the two-sided analyses was 95%. The chi-square test was used for comparing categorical variables, whereas the Mann-Whitney U test was used for comparing continuous variables. Logistic regression was used to assess the variables associated with the target case for *P. aeruginosa*, and crude ORs were calculated. A two-sided P-value of <0.05 was considered statistically significant.

## Results

**Figure 1:**
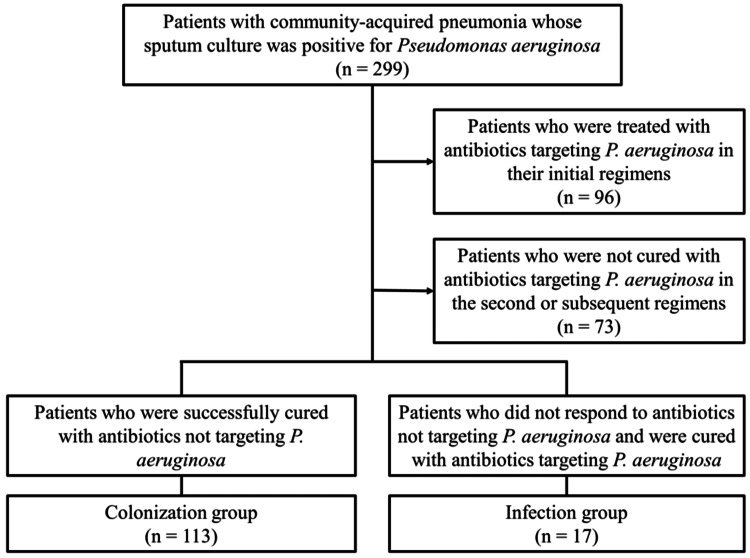
A flowchart of the participants.

Of 299 patients with CAP whose sputum culture was positive for *P. aeruginosa* during the study period, 203 (68%) were treated with antibiotics not targeting *P. aeruginosa* in their initial regimen (Figure 1). Regimens targeting *P. aeruginosa* included TAZ/PIPC (n = 67, 69.8%), PIPC (n = 12, 12.5%), LVFX (n = 8, 8.3%), MEPM (n = 5, 5.2%), and others (n = 4, 4.2%), whereas regimens not targeting *P. aeruginosa *included sulbactam/ampicillin (n = 158, 78%), ceftriaxone (n = 24, 12%), antibiotics to which *P. aeruginosa* was resistant (n = 11, 5.0%), vancomycin (n = 3, 1.5%), and others (n = 7, 3.5%). The group receiving antibiotics targeting *P. aeruginosa* had significantly higher ALT levels (median 17, interquartile range 12-28 mg/dL vs. 15, 11-23, p = 0.031) and lower serum albumin levels (median 2.9, interquartile range 2.5-3.3 g/dL vs. 3.1, 2.9-3.4, p = 0.006) than the group receiving antibiotics not targeting *P. aeruginosa* (Table 1). Mortality within 28 days after admission in the group treated with antibiotics targeting *P. aeruginosa* was significantly higher than that in the group treated with antibiotics not targeting *P. aeruginosa* (19% vs. 7%, p = 0.003). Other variables including sex, age, systolic blood pressure, presence of respiratory failure, and underlying diseases did not significantly differ between the groups. The proportions of coisolation of bacteria other than *P. aeruginosa* (65% vs. 68%, p = 0.504) and prior isolation of *P. aeruginosa* (46% vs. 40%, p = 0.332) were comparable between these groups.

**Table 1:** Clinical characteristics of patients treated with antibiotics covering and non-covering Pseudomonas aeruginosa in their first regimen. The chi-square test was used for categorical variables, and continuous variables were compared using the Mann–Whitney U test. ALT, alanine aminotransferase; BMI, body mass index; BUN, blood urea nitrogen; COPD, chronic obstructive pulmonary disease; CRP, C-reactive protein; eGFR, estimated glomerular filtration rate; interquartile range, IQR; WBC, white blood cells.

	P. aeruginosa coverage (n = 96)	Non-P. aeruginosa coverage (n = 203)	P-value
Female, number (%)	33 (34)	71 (35)	0.919
Male, number (%)	63 (66)	132 (65)	0.919
Age (years), median (IQR)	85 (76–90)	85 (77–90)	0.946
BMI (kg/m^2^), median (IQR)	17.7 (15.7–19.9)	18.2 (15.7–19.9)	0.248
Systolic blood pressure, (mmHg), median (IQR)	116 (102–131)	120 (109–138)	0.089
Impaired consciousness, number (%)	80 (83)	152 (75)	0.116
Respiratory failure, number (%)	58 (60)	107 (53)	0.117
WBC (/μL), median (IQR)	10,430 (7,428–14,330)	10,190 (7,030–13,460)	0.232
Neutrophil (/μL), median (IQR)	9,092 (5,858–12,266)	8,107 (5,172–11,547)	0.150
Hemoglobin (g/dL), median (IQR)	11.9 (10.7–13.6)	12.0 (10.7–13.1)	0.889
Albumin (g/dL), median (IQR)	2.9 (2.5–3.3)	3.1 (2.9–3.4)	0.006
ALT (IU/L), median (IQR)	17 (12–28)	15 (11–23)	0.031
BUN (mg/mL), median (IQR)	18.8 (15.9–26.2)	21.6 (16.0–28.8)	0.120
eGFR (mL/min/1.73m^2^), median (IQR)	81.2 (59.4–108.0)	76.0 (46.7–100.6)	0.130
CRP (mg/dL), median (IQR)	8.5 (3.3–15.1)	6.5 (2.9–12.1)	0.091
COPD, number (%)	13 (14)	30 (15)	0.776
Cardiac disease, number (%)	35 (36)	61 (30)	0.268
Diabetes mellitus, number (%)	20 (21)	35 (17)	0.454
28-day mortality, number (%)	18 (19)	15 (7)	0.003
Co-isolation of other bacteria, number (%)	62 (65)	139 (68)	0.504
Prior isolation of P. aeruginosa, number (%)	44 (46)	81 (40)	0.332

After excluding 73 of 203 patients who were not cured with antibiotics targeting *P. aeruginosa* in the second or subsequent regimens, 130 patients were finally included. Among 130 patients treated with antibiotics not targeting *P. aeruginosa* in their initial regimen, 17 were included in the infection group and 113 were included in the colonization group. Systolic blood pressure in the infection group was significantly lower than that in the colonization group (odds ratio, 0.971; 95% CI, 0.946-0.996; p = 0.026) (Table 2). Sensitivity and specificity were 62% and 71 %, respectively, when 118 mmHg of systolic blood pressure was considered a cut-off value. WBC (11,320, 10,230-15,040/μL vs. 9,539, 7,030-12,560/μL) and neutrophil (9,605, 7,203-11,100/μL vs. 7,615, 5,148-10,969/μL) counts in the infection group were higher than those in the colonization group, albeit not statistically significant. No other significant differences in sex, age, and presence of respiratory failure were observed between the groups. The proportions of coisolation of bacteria other than *P. aeruginosa *(68% vs. 82%, p = 0.611) and prior isolation of *P. aeruginosa* (45% vs. 41%, p = 0.760) were comparable between the two groups. Coisolated bacteria did not significantly differ between the two groups: MRSA (n = 20, 27%), methicillin-susceptible *S. aureus* (MSSA) (n = 13, 12%), *Klebsiella pneumoniae* (n = 13, 12%), and others in the colonization group and MRSA (n = 3, 18%), MSSA (n = 5, 29%), and *Hemophilus influenzae* (n = 3, 18%) in the infection group.

**Table 2:** Factors associated with Pseudomonas aeruginosa infection cases. Logistic regression was used to assess the variables associated with the target case for *P. aeruginosa*. ALT, alanine aminotransferase; BMI, body mass index; BUN, blood urea nitrogen; COPD, chronic obstructive pulmonary disease; CRP, C-reactive protein; eGFR, estimated glomerular filtration rate; interquartile range, IQR; WBC, white blood cells.

	Colonization cases (n = 113)	Infection case (n = 17)	Crude odds ratio	P-value
Female, number (%)	35 (31)	10 (59)	1.267 (0.516–3.114)	0.606
Male, number (%)	78 (69)	7 (41)	1.267 (0.516–3.114)	0.606
Age (years), median (IQR)	85 (77–90)	81 (75–90)	0.990 (0.938–1.044)	0.702
BMI (kg/m^2^), median (IQR)	18.6 (16.2–20.7)	17 (16.2–18.9)	0.906 (0.765–1.072)	0.249
Systolic blood pressure (mmHg), median (IQR)	123 (111–140)	114 (99–119)	0.971 (0.946–0.996)	0.026
Impaired consciousness, number (%)	81 (72)	14 (82)	1.844 (0.496–6.849)	0.361
Respiratory failure, number (%)	58 (51)	9 (53)	1.067 (0.384–2.962)	0.901
WBC (/μL), median (IQR)	9,530 (7,030–12,560)	11,320 (10,230–15,040)	1.000 (1.000–1.000)	0.062
Neutrophil (/μL), median (IQR)	7,615 (5,148–10,969)	9,605 (7,203–11,100)	1.000 (1.000–1.000)	0.071
Hemoglobin (g/dL), median (IQR)	12.0 (11.0–13.1)	12.3 (10.9–13.7)	1.080 (0.828–1.408)	0.571
Albumin (g/dL), median (IQR)	3.1(2.8–3.5)	3.2 (2.9–3.6)	1.374 (0.530–3.564)	0.513
ALT (IU/L), median (IQR)	15 (10–23)	17 (14–24)	1.017 (0.985–1.050)	0.305
BUN (mg/mL), median (IQR)	21.0 (15.3–28.9)	18.1 (16.2–20.9)	0.999 (0.961–1.039)	0.971
eGFR (mL/min/1.73m^2^), median (IQR)	80.2 (47.9–111.2)	78.2 (52.2–88.6)	0.992 (0.980–1.005)	0.230
CRP (mg/dL), median (IQR)	5.46 (2.43–10.9)	6.11 (3.14–10.4)	0.977 (0.892–1.069)	0.609
COPD, number (%)	19 (17)	2 (12)	0.660 (0.139–3.125)	0.600
Cardiac diseases, number (%)	37 (33)	3 (18)	0.440 (0.119–1.627)	0.219
Diabetes mellitus, number (%)	19 (17)	3 (18)	1.060 (0.277–4.052)	0.932
Glucocorticoid use, number (%)	9 (8)	3 (18)	2.476 (0.598-10.25)	0.211
Number of P. aeruginosa colonies, number (%)				
0	9 (8)	1 (6)	0.998 (0.497-2.003)	0.996
+1	63 (56)	11 (65)		
+2	33 (29)	3 (18)		
+3	8 (7)	14 (82)		
Co-isolation of other bacteria, number (%)	77 (68)	14 (82)	1.308 (0.465–3.677)	0.611
Prior isolation of P. aeruginosa, number (%)	51 (45)	7 (41)	0.851 (0.302–2.394)	0.760

## Discussion

In this study, systolic blood pressure in the infection group was significantly lower than that in the colonization group. *P. aeruginosa* infection produces proinflammatory cytokines more acutely than other bacterial infections, which may lead to systemic vasodilatation [21,22]. For example, IL-10, IFN-γ, and IL-4 levels in patients in whom *P. aeruginosa* was isolated were significantly higher than those in whom *S. aureus* was isolated [22]. *P. aeruginosa* infection is also a risk factor for rapid deterioration in a patient’s clinical condition [23]. A review revealed that* P. aeruginosa* CAP is often rapidly progressive and can occur in previously healthy individuals with a 33% mortality rate, suggesting that *P. aeruginosa *must be considered in the differential diagnosis for anyone presenting with a rapidly progressive pneumonia, particularly in those with a history of smoking; moreover, detection of Gram-negative bacilli in the sputum should raise clinical diagnostic suspicion [23]. The results of the present study are likely to be consistent with these phenomena. However, the systolic blood pressure in the infection group remained in the normal range, although it was lower than that in the colonization group. This finding could be a clue to distinguishing between infection and colonization, but its implementation in clinical practice remains challenging. Furthermore, information regarding hypertension and hypotension as an underlying disease and the control status should be considered.

In contrast, high CRP and low albumin levels are associated with disease severity among patients with pneumonia [24-26], but no significant differences were observed between the infection and colonization groups in the present study. When these markers were compared between patients treated with antibiotics targeting and not targeting *P. aeruginosa*, the group receiving antibiotics targeting *P. aeruginosa* had significantly lower serum albumin levels than the group receiving antibiotics not targeting *P. aeruginosa*, and the 28-day mortality in the group treated with antibiotics targeting *P. aeruginosa* was significantly higher than that in the other group. These results indicate that CRP and albumin levels could be useful for predicting a clinical prognosis but not differentiating isolated *P. aeruginosa* that needs to be targeted from *P. aeruginosa* that does not need to be targeted among patients with pneumonia.

Although some studies have suggested that the number of isolated bacteria in a respiratory sample can aid in distinguishing infection from colonization [12,27], no significant difference in this number was observed between the groups in this study. The number of isolated bacteria is generally assessed using colony-forming units (CFUs) [28]. CFU provides a direct measure of viable bacterial cells and refers to the number of individual colonies of any microorganism grown on a plate of media. The present study assessed the number of CFUs using a 4-point scale. Any discrepancy in the assessment procedure might have affected the results.

The strength of this study was that cases in which isolated *P. aeruginosa* had to be targeted or not were defined based on the clinical course of antibiotic responses. This is more likely to provide an accurate differentiation between infection and colonization than other surrogate approaches, such as the presence of phagocytized bacteria observed through Gram staining or the number of CFUs. However, this study had some limitations. First, selection bias might have existed due to the retrospective nature of this study. For example, this study focused on patients who were treated with antibiotics not targeting *P. aeruginosa* in their first regimen; therefore, those who were initially treated with antibiotics specific to *P. aeruginosa* were excluded. Indeed, patients who were treated with antibiotics targeting *P. aeruginosa* had higher ALT levels, lower serum albumin levels, and higher 28-day mortality than those treated with antibiotics not targeting *P. aeruginosa*. This may have resulted in the exclusion of patients with severe pneumonia. Thus, the difference in clinical manifestation between the colonization and infection groups might have been underestimated. Second, we defined an infection case as a patient who did not respond to antibiotics not targeting *P. aeruginosa* but was cured with antibiotics targeting *P. aeruginosa*. The possibility that antimicrobial-resistant bacteria other than *P. aeruginosa* were killed by the administration of the second regime cannot be excluded. Extended-spectrum β-lactamase bacteria, β-lactamase-negative ampicillin-resistant *H. influenzae,* or penicillin-resistant *Streptococcus pneumoniae *are potential pathogens. However, these bacteria generally account for only a small percentage of the causative microorganisms of pneumonia among older people [29,30]; thus, this effect was less likely to impact our results. Third, this study did not evaluate sputum quality and phagocytic features on the Gram staining, which could have helped in determining whether isolated pathogens were infection or colonization. Finally, because the sample size was small, despite multiple institutional studies, multivariate analysis could not be performed. Cofounding factors with systolic blood pressure need to be considered, such as laboratory data possibly including cytokine levels.

## Conclusions

Decreased systolic blood pressure might serve as a useful marker for distinguishing isolated *P. aeruginosa* that needs to be targeted from *P. aeruginosa* which does not need to be targeted among patients with CAP in whom the bacteria were isolated from respiratory samples. However, as mentioned in the limitation section, the study population may have a severe case exclusion, which might have resulted in a slight difference in systolic blood pressure and may have been confounded by other clinical factors. The differential diagnosis between colonization and infection may contribute to the proper use of antibiotics in the treatment of patients with pneumonia. It would be reasonable to conduct differential assessments in patients with HAP because *P. aeruginosa* is also a major pathogen in HAP. A large-scale, observational study or a randomized, controlled trial is required to validate the results of this study.
